# Mutation screening and haplotype analysis of the rhodopsin gene locus in Japanese patients with retinitis pigmentosa

**Published:** 2007-06-29

**Authors:** Yuichiro Ando, Masayuki Ohmori, Hideki Ohtake, Kuniyo Ohtoko, Shigeru Toyama, Ron Usami, Aya O’hira, Hiromi Hata, Kenji Yanashima, Seishi Kato

**Affiliations:** 1 Department of Rehabilitation Engineering, Research Institute, National Rehabilitation Center for Persons with Disabilities, Saitama, Japan; 2 Department of Applied Chemistry, Graduate School of Engineering, Toyo University, Saitama, Japan; 3 Department of Ophthalmology, Hospital, National Rehabilitation Center for Persons with Disabilities, Saitama, Japan

## Abstract

**Purpose:**

To identify nucleotide sequence variations in the rhodopsin (RHO) gene of Japanese patients with retinitis pigmentosa (RP) in order to search for mutations or haplotypes responsible for RP.

**Methods:**

The entire region of *RHO* locus including a promoter region and introns was sequenced using blood-derived genomic DNA samples donated by 68 patients with RP and 68 control subjects.

**Results:**

We found 39 single nucleotide substitutions including 17 rare substitutions of less than 1% in frequency, one insertion/deletion polymorphism, and one CA-repeat polymorphism in a 7.8 kbp region spanning the promoter, five exons, and four introns of the *RHO* gene locus. There were no affected subjects with amino acid substitutions in RHO, and there was 1 control subject with a novel substitution (Ala42Thr) who had no symptoms of RP. Fine analysis of single nucleotide polymorphism (SNPs) revealed eight haplotype structures of the Japanese *RHO* locus. There was no significant difference between RP patients and controls in terms of haplotype frequency.

**Conclusions:**

No mutation causing an amino acid substitution of RHO was observed in 68 Japanese patients with RP, but 1 control subject did have a novel amino acid substitution. The Japanese *RHO* locus is comprised of eight major haplotypes. The RP-associated haplotype was not identified. The haplotype-tagging SNPs identified in this study will be useful as markers for the linkage-based screening of RP patients.

## Introduction

Retinitis pigmentosa (RP) is a group of hereditary retinal diseases characterized by night blindness and progressive loss of the visual field. It has a prevalence of about 1 in 4000 [1]. RP was the major cause of visual impairment among persons who entered 29 rehabilitation centers in Japan, accounting for 25% of these cases [2]. RP is a disease with considerable genetic heterogeneity; it is transmitted as an autosomal dominant, autosomal recessive, X-linked, or rare mitochondrial form. Moreover, digenic or syndromic forms of RP complicate inheritance patterns [3,4]. The frequency of the inherited type has been estimated as follows: 19% dominant, 65% recessive or isolated, 8% X-linked, and 8% undetermined in Maine in the United States [5]; 16.9% dominant, 25.2% recessive, 1.6% X-linked, and 56.3% simplex in a study of Japanese RP patients [6].

So far, positional cloning and candidate gene screening have identified at least 36 loci responsible for RP [3] (for the latest summary, see the RetNet page). Of the genes known to cause autosomal dominant RP (adRP) in the United States and Canada, mutations in a rhodopsin (RHO) gene were most frequently observed, accounting for 24-29% of cases [7-9]. Since *RHO* was identified as an RP-causing gene [10], a number of mutations have been found at a variety of sites on the *RHO* locus [1,7-9] (for the latest summary, see RHO). Most rhodopsin mutations cause adRP, whereas a few have been reported to cause autosomal recessive RP (arRP) [11,12] or congenital stationary night blindness [13-15]. In a total of 85 Japanese adRP patients, the following five causative mutations in RHO have been reported: Asn15Ser [16], Thr17Met [17], Gly106Arg [18], Glu181Lys [19], Pro347Leu [17]. The rate of RHO mutations in Japanese (5/85, 5.9%) is lower than that in the United States, suggesting a race-dependent mutation rate [20].

A single-stranded conformation polymorphism method targeting the coding regions has been frequently used for the first screening of mutations [7-9,11], but this method intrinsically includes the possibility of missing mutations. Mutations in a promoter region or introns may also be responsible for arRP by affecting the levels of expression of *RHO* [13]. These problems with mutation screening can be resolved by sequencing the entire region of the *RHO* locus. Furthermore, full sequencing provides information about single nucleotide polymorphism (SNPs) and haplotypes that could affect the expression levels of the gene. Promoter haplotype combinations have been shown to affect the biological and therapeutic phenotypes by altering the expression of the gene for β2-adrenergic receptor [21]. This led us to hypothesize that *RHO* promoter haplotypes are associated with susceptibility to RP via a mechanism that changes the levels of expression of *RHO*. A search for RP-related SNPs in *RHO* was carried out [22], but there has been no report on the haplotype structure of *RHO* to date.

In the present study, we determined the full sequence of the *RHO* locus, including the promoter regions and introns, in order to search for RP-associated mutations and/or haplotypes in the *RHO* loci of RP patients who visited the low-vision clinic at our center.

## Methods

### Subjects

We studied 68 unrelated RP patients who visited the low-vision clinic at our center. RP subjects were selected on the basis of clinical findings, patient history, and family history. Of these 68 patients, 26 had a family history, 27 had no family history, and 15 had an unknown family history. Table 1 shows the detailed information on the 26 subjects with a family history. Five of these patients had an affected parent with RP or night blindness, and the patient designated as RP048 reported having an affected sibling whose son suffers from RP. Thus, these six cases were diagnosed as adRP. Seven patients with grandparents or cousins with RP or night blindness could have suffered from adRP. RP051 and RP062 reported a family history of consanguineous marriage. Control samples were obtained from students at the college affiliated with our center and from members of our staff of the center, all of whom declared having neither a personal history nor a family history of night blindness or unexplained visual loss. The study was approved by the Human Genome-Ethics Committee of the National Rehabilitation Center for Persons with Disabilities and was performed in accordance with the Declaration of Helsinki. Informed consent was obtained from all subjects involved in this study.

**Table 1:** Information on retinitis pigmentosa patients with a family history.

**Number**	**Patient ID**	**Sex**	**Number of siblings**	**Number of affected siblings**	**Other affected relatives**	**Type of inheritance**
1	RP005	F	6	2	Mother (RP)	ad
2	RP008	F	7	2		
3	RP009	F	4	2	Grandmother (NB)	ad(?)
4	RP010	F	4	3		
5	RP016	F	8	3	Grandmother (RP?)	ad(?)
6	RP017	F	5	2		
7	RP020	F	3	1	Cousin (central vision loss)	ad(?)
8	RP021	M	3	2		
9	RP022	M	3	1	Grandfather (RP?)	ad(?)
10	RP023	M	7	2		
11	RP024	M	2	2	Grandmother (RP?)	ad(?)
12	RP029	M	3	2	Mother (NB), cousin (RP)	ad
13	RP034	F	5	1	Father (NB), cousin (RP?)	ad
14	RP035	M	3	1	Father (RP)	ad
15	RP036	F	8	5	Distant relative (RP?)	ad(?)
16	RP037	F	3	1	Mother(RP)	ad
17	RP038	F	4	1	Cousin(RP)	ad(?)
18	RP039	M	2	2		
19	RP044	F	6	2		
20	RP045	M	10	4		
21	RP048	M	4	3	Affected sib's son (RP)	ad
22	RP051	F	2	2	Consanguineous marriage	ar
23	RP061	M	4	2		
24	RP062	M	4	2	Consanguineous marriage	ar
25	RP063	M	6	3		
26	RP064	F	7	3		

Of 68 unrelated RP patients who visited the low-vision clinic at our center, 26 had a family history. Sex: Male (M), Female (F). Siblings and affected siblings include the proband. NB indicates night blindness. Inheritance type: ad indicates autosomal dominant; ar indicates autosomal recessive.

### DNA sample preparation

Genomic DNA was isolated from venous blood using a PUREGENE DNA purification Kit (Gentra Systems, Minneapolis, MN). A sample for DNA sequencing was prepared by polymerase chain reaction (PCR) using genomic DNA as a template. The genomic sequence of the *RHO* locus (NT_005612.15) was retrieved from NCBI database (Build 36.1). Nucleotide A of the initiation codon of *RHO* was defined as Position 1. The primer sequences for PCR amplification are listed in Table 2. The first PCR amplification was performed in order to obtain two amplicons, A1 (containing *RHO* exons and introns) and A2 (containing the promoter region of *RHO*). Two hundred nanograms of genomic DNA were amplified with Taq polymerase (TaKaRa Ex Taq® Takara Bio Inc., Shiga, Japan) in a 50 μl reaction volume for 30 cycles under conditions of heating for 30 s at 96 °C, annealing for 30 s at 63 °C, and extension for 2 min at 72 °C. The second PCR amplification using amplicon A1 as a template produced 4 amplicons (A3, A4, A5, A6).

**Table 2:** Polymerase chain reaction primers used for amplifying the *rhodopsin* gene locus.

**Amplicon**	**Primer**	**Position**	**Sequence (5'->3')**
A1	A1F	-297	aaggccgcctcggCCTGGATCCTGAGTACCTCTCCTC
	A1R	6605	aagcggccgcTTTTCCCATTCCCAGGACTGCCTCCTCCAC
A2	A2F	-1260	CAGTCATCTGCCCCCAAGGC
	A2R	142	TCAGCAGAAACATGTAGGCG
A3	A1F	-297	aaggccgcctcggCCTGGATCCTGAGTACCTCTCCTC
	A3R	2402	CCTGGAACCAGACACTACTG
A4	A4F	1951	GCTCTCCTCAGCGTGTGGTC
	A4R	4669	GCTGTGTCACCCGTGACATTTCAT
A5	A5F	3735	CATGCATCTGCGGCTCCTGCTC
	A5R	6010	CTTCCAGAGGCTGAGAGAAAGGCC
A6	A6F	5085	GAACGAAGTCACATAGGCTCC
	A1R	6605	aagcggccgcTTTTCCCATTCCCAGGACTGCCTCCTCCAC

Position 1 corresponds to the nucleotide A of the initiation codon of the RHO gene. Lower letters represent an artificial sequence added for cloning the polymerase chain reaction products.

### Sequencing

PCR products were sequenced bi-directionally with a BigDye® Terminator Cycle Sequencing Kit (Applied Biosystems, Foster City, CA) using the primers listed in Table 3. Sequencing reaction products were run on an automated capillary sequencer (Applied Biosystems 3130xl Genetic Analyzer, Applied Biosystems, Foster City, CA).

**Table 3:** Primers used for sequencing.

**Primer**	**Position**	**Sequence (5'-3')**
**Forward**		
SF01	-926	CTAGCGTTCAAGACCCATTAC
SF02	-452	GAGAGACTGGGAGAATAAACC
SF03	153	CTTCCCCATCAACTTCCTCACG
SF04	592	TGCGCTTGTCTAATTTCACAGC
SF05	1053	GGAAAACAGATGGGGTGCTGC
SF06	1476	CTTTCACTGTTAGGAATGTCC
SF07	1951	GCTCTCCTCAGCGTGTGGTC
SF08	2383	CAGTAGTGTCTGGTTCCAGG
SF09	2860	CCTCCTCAGTCTTGCTAGGGTC
SF10	3289	GGTGTCATCTGGTAACGCAG
SF11	3735	CATGCATCTGCGGCTCCTGCTC
SF12	4144	TGGCAGCAGTCTTGGGTCAGC
SF13	4616	ACAGAACACCCTTGGCACACAGAG
SF14	5085	GAACGAAGTCACATAGGCTCC
SF15	5360	GATGGATGCAGGAAGGAATGGAGG
SF16	5548	CTGAGAAGACCAAAAGAGGTG
SF17	5987	GGCCTTTCTCTCAGCCTCTGGAAG
SF18	6444	GGGTTTTGTTGCTTTCACACTC
**Reverse**		
SR01	-775	GTGGCTGTGAGGTTGTGGAGAC
SR02	-284	ACTCAGGATCCAGGAAAAGG
SR03	174	CGTGAGGAAGTTGATGGGGAAG
SR04	613	GCTGTGAAATTAGACAAGCGCA
SR05	1073	GCAGCACCCCATCTGTTTTCC
SR06	1496	GGACATTCCTAACAGTGAAAG
SR07	1970	GACCACACGCTGAGGAGAGC
SR08	2402	CCTGGAACCAGACACTACTG
SR09	2881	GACCCTAGCAAGACTGAGGAGG
SR10	3308	CTGCGTTACCAGATGACACC
SR11	3756	GAGCAGGAGCCGCAGATGCATG
SR12	4204	GCCAGGAATCTGCATTTCTCAC
SR13	4669	GCTGTGTCACCCGTGACATTTCAT
SR14	5105	GGAGCCTATGTGACTTCGTTC
SR15	5568	CACCTCTTTTGGTCTTCTCAG
SR16	6010	CTTCCAGAGGCTGAGAGAAAGGCC
SR17	6465	GAGTGTGAAAGCAACAAAACCC

Position 1 corresponds to the nucleotide A of the initiation codon of the *RHO* gene.

### Haplotype analysis

Haplotypes were estimated from unphased genotypes using Clark's algorithm [23] and the expectation-maximization (EM) algorithm [24]. Phylogenetic analysis was carried out with the minimum spanning network (MSN) algorithm [25]. The Arlequin program (Schneider, Roessli, Excoffier, Arlequin version 2.000: A software for population genetics data analysis, Geneva, Switzerland) was used for the analysis using the EM algorithm and the MSN algorithm.

## Results

### Sequence variations

Table 4 shows the sequence variations obtained in this study. We identified a total of 41 sequence variations consisting of 39 single nucleotide substitutions, one insertion/deletion polymorphism (Indel, SV15), and one CA-repeat polymorphism (SV16) in a total of 272 chromosomes from 68 RP patients and 68 controls. Of 39 single nucleotide substitutions, 25 sites were novel ones not registered in the dbSNP database (National Center of Biotechnology Information). This study did not find four substitutions (rs2855553, rs2855556, rs2625964, rs2625969) registered in the dbSNP, which seem to be rare among Japanese. Rare substitutions with less than 1% minor allele frequency (MAF) occurred at 17 sites, nine of which were observed only in the RP patients and six only in the controls.

**Table 4:** Nucleotide sequence variations observed in the *rhodopsin* gene locus.

							**Minor allele frequence (%)**				
**Marker name**	**SNP name**	**Region**	**Amplicon**	**Position**	**Alleles major/minor**	**dbSNP ID**	**RP**	**Ctl**	**Sum**	**JSNP**	**JSNP ID**
SV01	SNP01	Upstream	A2	-1163	C/T	rs2625954	42.6	41.9	42.3	-	-
SV02	SNP02	Upstream	A2	-778	A/C	rs2625955	41.9	41.9	41.9	42	IMS-JST108944
SV03	-	Upstream	A2	-598	G/A	-	0.7	0	0.4	-	-
SV04	SNP03	Exon 1	A2, A3	-51	G/A	rs2269736	33.8	28.7	31.3	33.2	IMS-JST024023
SV05	SNP04	Exon 1	A2, A3	-26	G/A	rs7984	43.4	41.2	42.3	41.2	IMS-JST024024
SV06	-	Exon 1	A2, A3	124	G/A(Ala42Thr)	-	0	0.7	0.4	-	-
SV07	-	Exon 1	A3	273	C/T(Phe91Phe)	-	0.7	0	0.4	-	-
SV08	-	Exon 1	A3	315	C/T(Phe105Phe)	-	0.7	0	0.4	-	-
SV09	SNP05	Intron 1	A3	668	A/G	-	5.9	3.7	4.8	-	-
SV10	SNP06	Intron 1	A3	709	C/T	-	5.9	5.1	5.5	-	-
SV11	-	Intron 1	A3	794	G/A	-	0	0.7	0.4	-	-
SV12	-	Intron 1	A3	966	C/T	-	0.7	0	0.4	-	-
SV13	SNP07	Intron 1	A3	1349	T/G	rs2855552	29.4	30.1	29.8	28.3	IMS-JST130259
SV14	-	Intron 1	A3	1354	C/T	-	1.5	1.5	1.5	-	-
-	-	Intron 1	-	1535	G/A	rs2855553	-	-	-	-	-
SV15	-	Intron 1	A3	1751-1760	AGGATGCATT/del	-	22.1	19.1	20.6	-	-
SV16	-	Intron 1	A3	1775-1835	(CA)nA(CA)m	-	-	-	-	-	-
SV17	SNP08	Intron 2	A4	2703	G/T	rs6803468	0.7	3.7	2.2	-	-
SV18	SNP09	Intron 2	A4	2718	C/T	-	5.9	5.1	5.5	-	-
SV19	SNP10	Intron 2	A4	2750	G/A	rs6803484	0.7	3.7	2.2	-	-
-	-	Intron 2	-	2760	A/T	rs2855556	-	-	-	-	-
SV20	-	Intron 2	A4	2753	A/G	-	0	0.7	0.4	-	-
SV21	-	Intron 2	A4	2787	G/A	-	1.5	0	0.8	-	-
SV22	SNP11	Intron 2	A4	2990	G/A	-	22.1	19.9	21	-	-
SV23	SNP12	Intron 2	A4	3217	C/T	-	5.9	5.1	5.5	-	-
SV24	-	Intron 2	A4	3269	C/T	-	0.7	0	0.4	-	-
SV25	-	Intron 2	A4	3397	G/A	-	0.7	0.7	0.7	-	-
SV26	SNP13	Intron 3	A4	3687	C/T	-	5.9	5.1	5.5	-	-
SV27	-	Exon 4	A4, A5	3994	C/T(Ser297Ser)	-	1.5	1.5	1.5	-	-
SV28	-	Intron 4	A4, A5	4056	G/T	-	1.5	0	0.8	-	-
SV29	SNP14	Intron 4	A4, A5	4346	T/A	rs2855557	41.9	42.6	42.3	-	-
-	-	Intron 4	-	4730	T/A	rs2625964	-	-	-	-	-
SV30	SNP15	Intron 4	A5	4852	G/A	rs2071092	29.4	30.1	29.8	28.8	IMS-JST006396
SV31	SNP16	Exon 5	A5	5028	C/A	rs2071093	5.9	5.1	5.5	4.3	IMS-JST006397
SV32	-	Exon 5	A5, A6	5216	C/T	-	0.7	0	0.4	-	-
SV33	SNP17	Exon 5	A5, A6	5217	G/A	rs2410	48.5	49.3	48.9	-	-
SV34	-	Exon 5	A5, A6	5426	G/A	-	0.7	0.7	0.7	-	-
-	-	Exon 5	-	5833	A/G	rs2625969	-	-	-	-	-
SV35	SNP18	Exon 5	A5, A6	5897	G/A	rs2855558	41.9	41.9	41.9	42.5	IMS-JST086767
SV36	SNP19	Exon 5	A5, A6	5910	T/C	-	0.7	2.9	1.8	-	-
SV37	-	Exon 5	A5, A6	5914	C/T	-	0	0.7	0.4	-	-
SV38	-	Exon 5	A6	5944	A/G	-	0.7	0	0.4	-	-
SV39	SNP20	Exon 5	A6	6084	G/A	rs3733148	8.8	19.9	14.4	15.1	IMS-JST086768
SV40	-	Exon 5	A6	6129	C/T	-	0	0.7	0.4	-	-
SV41	-	Exon 5	A6	6193	G/A	rs3733149	0	0.7	0.4	0.8	IMS-JST086769

The amino acid substitution caused by nucleotide substitution was represented in parenthesis. Individual SNPs were characterized in 68 patients (RP) and 68 controls (Ctl). A database of Japanese single nucleotide polymorphisms (JSNP) contains nine SNPs that were measured in 732-746 Japanese subjects.

Twenty substitutions with a MAF of greater than 1% showed linkage disequilibrium (LD) between pairs, and these substitutions were designated as SNP01 to SNP20. These SNPs were consistent with the Hardy-Weinberg equilibrium (data not shown). SV14 and SV27 exhibited 1.5% of the MAF, but were excluded from the SNPs due to the absence of LD pairs. Most SNPs with a MAF of greater than 10% were registered in the dbSNP, with the exception of SNP11. Nine SNPs were registered in a database of Japanese single nucleotide polymorphisms (JSNP) and the MAF values of these substitutions were similar to each other at each site, except in the case of SNP20. The differences between RP patient and control MAFs were small, except in the case of SNP20. The MAF at SNP20 was 8.8% among RP patients and 19.9% among controls, respectively. However, the mean MAF of all subjects at SNP20 was 14.4%, which is similar to the value described in the JSNP database, i.e., 15.1% (1488 chromosomes).

Four rare single nucleotide substitutions were observed in the coding region. Three substitutions (SV07, SV08, and SV27) were synonymous. On the other hand, SV06 observed in 1 control led to an amino acid substitution (Ala42Thr). SV03 was observed in the promoter region of one RP patient, and it was located at position -503 upstream of the transcription start site [26].

Intron 1 was found to contain an Indel of 10bp (SV15) followed by a CA-repeat polymorphism (SV16, (CA)nA(CA)m, n=12-14, m=15-27). The precise number of repeats was not determined due to degeneration of sequences.

### Haplotypes

Haplotypes of the *RHO* locus were estimated on the basis of 20 SNPs of 272 chromosomes obtained from patients and controls. Indel variation SV15 was also taken into account, because it was found to be linked to SNP11. Table 5 shows 14 haplotypes consisting of eight major haplotypes and six rare ones. The rare haplotypes (H09-H14) appeared to be the result of a single conversion in the corresponding major haplotype. There were five groups of LD structures of SNPs and an Indel: SNP01-SNP02-SNP04-SNP14-SNP18, SNP06-SNP09-SNP12-SNP13-SNP16, SNP07-SNP15, SNP08-SNP10-SNP19, and SNP11-SV15. Eight major haplotypes can produce 36 combinations, in which 29 were identified in 136 subjects. Seven major haplotypes, except for H08, were found to exist as a homozygote. The most frequent haplotype, H01, accounted for 27.2% of a total of 272 chromosomes. The present comparison of haplotype frequencies did not reveal any significant differences between RP patients and controls when taking into account the multiple comparisons. With a Bonferroni correction for the eight comparisons, a p-value of 0.05/8=0.006 is required for statistical significance.

**Table 5:** Haplotypes of the Japanese *rhodopsin* gene locus.

**Haplotype name**	**SNP name**																					**Number of chromosomes**				**χ^2^**	**p value**
	1	2	3	4	5	6	7	Indel	8	9	10	11	12	13	14	15	16	17	18	19	20	RP	Ctl	Sum	Frequency		
H01	T	C	G	A	A	C	T	Ins	G	C	G	G	C	C	A	G	C	A	A	T	G	45	29	74	0.272	4.75	0.029
H02	C	A	A	G	A	C	T	Del	G	C	G	A	C	C	T	G	C	G	G	T	G	30	26	56	0.206	0.36	0.548
H03	T	C	G	A	A	C	T	Ins	G	C	G	G	C	C	A	G	C	A	A	T	A	12	27	39	0.143	6.73	0.009
H04	C	A	G	G	A	C	G	Ins	G	C	G	G	C	C	T	A	C	G	G	T	G	16	23	39	0.143	1.47	0.225
H05	C	A	A	G	A	C	G	Ins	G	C	G	G	C	C	T	A	C	G	G	T	G	15	13	28	0.103	0.16	0.69
H06	C	A	G	G	A	T	T	Ins	G	T	G	G	T	T	T	G	A	A	G	T	G	8	6	14	0.051	0.3	0.583
H07	C	A	G	G	G	C	G	Ins	G	C	G	G	C	C	T	A	C	G	G	T	G	8	4	12	0.044	1.39	0.238
H08	C	A	G	G	A	C	T	Ins	T	C	A	G	C	C	T	G	C	A	G	C	G	1	3	4	0.015	1.01	0.313
H09	T	C	G	G	A	C	T	Ins	G	C	G	G	C	C	A	G	C	A	A	T	G	0	1	1	0.004		
H10	T	A	A	G	A	C	G	Ins	G	C	G	G	C	C	T	A	C	G	G	T	G	1	0	1	0.004		
H11	C	A	G	G	A	T	G	Ins	G	T	G	G	T	T	T	G	A	A	G	T	G	0	1	1	0.004		
H12	C	A	G	G	G	C	T	Ins	G	C	G	G	C	C	T	A	C	G	G	T	G	0	1	1	0.004		
H13	C	A	G	G	A	C	T	Ins	T	C	A	G	C	C	A	G	C	A	G	C	G	0	1	1	0.004		
H14	C	A	G	G	A	C	T	Ins	T	C	A	G	C	C	T	G	C	A	G	T	G	0	1	1	0.004		
																					Total	136	136	272	1.000		

A total of 136 subjects comprised of 68 retinitis pigmentosa patients (RP) and 68 controls (Ctr) were analyzed. One insertion/deletion (Indel) variation was linked to SNP11. Red letters represent haplotype-tagging SNPs.

### Phylogeny of *rhodopsin* gene haplotypes

**Figure 1:**
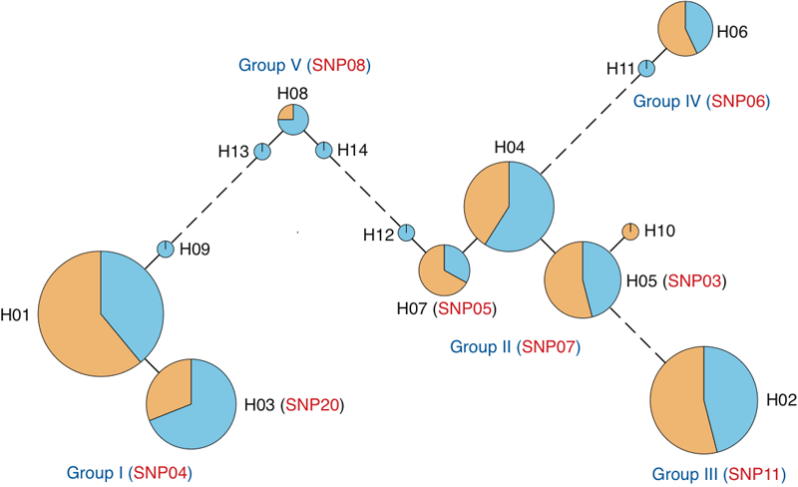
Phylogeny of Japanese *rhodopsin* gene haplotypes. The area of the circle is proportional to the frequency of each haplotype. The ratio of RP patients (orange) and controls (light blue) for each haplotype is color-coded. The number of line segments between circles corresponds to the number of nucleotide substitutions between haplotypes. The *RHO* haplotypes can be classified into five groups designated as Group I to Group V. The haplotype-tagging SNP (htSNP) distinguishing each group is described in the parentheses following the group name and the haplotype name.

The phylogeny of *RHO* haplotypes was calculated using the minimum spanning network algorithm (Figure 1). The area of the circle is proportional to the frequency of each haplotype. The ratio of RP patients (orange) and controls (light blue) for each haplotype is color-coded. The number of line segments between circles corresponds to the number of nucleotide substitutions between haplotypes. The graph in Figure 1 shows that the *RHO* haplotypes can be classified into five groups separated by a large number of nucleotide substitutions. The groups are designated as Group I to Group V. The haplotype-tagging SNP (htSNP) distinguishing each group is described in the parenthesis following the group name in the Figure 1. Furthermore, the htSNP distinguishing each haplotype in the group is described in parenthesis following the haplotype name. These htSNPs can be used as markers to estimate the haplotype of new subjects.

## Discussion

In this study, we did not detect any causative mutation in the coding region of the RHO gene of 68 Japanese RP patients. Five mutations in RHO have been reported to date for a total of 85 Japanese adRP patients [16-19]; thus, the prevalence of causative mutations in RHO is calculated to be 5.9%, suggesting that RHO mutations may be among the common causes of adRP in Japan. Based on the estimated number (at most 13) of adRP patients in this study, the predicted number of cases with a RHO mutation was less than 1. Thus, the present results are consistent with those of previous studies, indicating that the rate of RHO mutations in Japanese adRP is low compared to that of the American population. It should be noted that more frequent occurrence of RHO mutations in the American patients is partially due to the presence of a large number of families carrying Pro23His [27], which likely originated from a single ancestor [28].

Unexpectedly, one novel heterozygous variation causative of an amino acid substitution (Ala42Thr) was found in 1 control subject. This variation exists in the first transmembrane domain. To date, eleven pathogenic variations have been reported in the first transmembrane domain of RHO. The Ala residue at position 42 is fairly conserved among vertebrate opsins. Of 143 reported vertebrate opsin sequences, 130 have an Ala at position 42, whereas 12 have Cys and 1 has Ser at that position (GPCRDB: Information system for G protein-coupled receptors (gpcr), release 10.0) [29]. The amino acid change from a nonpolar Ala to a polar and slightly larger Thr could affect the conformation of the first transmembrane domain. A similar substitution close to the 42nd Ala, Met44Thr, has been reported in a patient with a simplex case of RP [30]. These findings suggest that Ala42Thr could be a pathogenic mutation. However, the control subjects reported having no symptoms such as night blindness or other visual impairments, nor was there any family history of visual impairments. There are three possibilities that might account for the discrepancy between the predicted pathogenic phenotype and the apparently silent phenotype of the carrier. The first possibility would be that Ala42Thr is a rare benign variation. In fact, four nonpathogenic missense changes have been reported thus far: Thr70Met [9], Val104Ile [31], Pro220Leu [27], and Gly284Ser [32]. On the other hand, a missense change, Glu150Lys, caused recessive RP [12], and this second possibility suggests that the Ala42Thr change might be a recessive mutation. The third possibility would be that the symptoms were sufficiently mild to escape the notice of the carrier. Unfortunately, this issue remains unsolved, as we were unable to obtain any clinical information on this subject.

Full sequencing of the *RHO* locus has revealed the presence of variations in the promoter region and introns. Seven heterozygous variations occurred only in the RP patients: 1 in the promoter region, 4 in the introns, and 2 in the 3'-UTR. Although we cannot deny the possibility of causative mutations, most cases may be rare variations. SV03 is located at position -503 upstream of the transcription start site. This region did not show significant homology with the bovine *RHO* promoter [33], and the corresponding bovine sequence was not included in the protected regulatory elements determined by DNase I footprint experiments [34]. We were also unable to identify regulatory elements for retina-specific transcription factors such as CRX, NRL, and NR2E3 [35] in this region. These results suggest that SV03 is unlikely to affect promoter activity. This issue should be resolved in future experiments by measuring the promoter activity of SV03 variants.

The fine mapping of SNPs has revealed eight haplotype structures of the *RHO* locus in the Japanese population. The haplotypes obtained are classified into five groups that may stem from distinct Japanese ancestors. If a causative mutation is associated with a specific haplotype, it may stem from a single ancestor. From this perspective, it would be of interest to determine which haplotypes carry frequent mutations such as Pro23His and Pro347Leu [8].

Unfortunately, we were unable to identify RP-associated SNPs or haplotypes. Our first expectation was that the haplotype of the promoter region may be associated with RP by altering the levels of expression of *RHO*. The promoter region contains five SNPs (SNP01-SNP05) that construct four haplotypes. Assessment of frequencies of these four haplotypes did not reveal any significant differences between RP patients and controls (data not shown), suggesting that there is no association between susceptibility to RP and the *RHO* promoter haplotype.

In conclusion, this was the first study to determine the comprehensive sequence variations and haplotype structure of the *RHO* locus, including the promoter region and introns. No causative mutation in *RHO* was observed in 68 Japanese patients with RP, but 1 control subject had a novel amino acid substitution, Ala42Thr, which may have been benign. Although we were unable to identify any RP-associated SNPs or haplotypes, the htSNPs identified here will be useful as markers for the linkage-based screening of RP patients.
